# The effect of heat treatment on the anatase–rutile phase transformation and photocatalytic activity of Sn-doped TiO_2_ nanomaterials

**DOI:** 10.1039/c8ra00766g

**Published:** 2018-04-17

**Authors:** Xiaodong Zhu, Shihu Han, Wei Feng, Qingquan Kong, Zhihong Dong, Chenxi Wang, Jiahao Lei, Qian Yi

**Affiliations:** College of Mechanical Engineering, Chengdu University Chengdu 610106 China fengwei1981_829@foxmail.com; College of Materials and Chemistry & Chemical Engineering, Chengdu University of Technology Chengdu 610059 China; College of Architecture and Environment, Sichuan University Chengdu 610065 China

## Abstract

Sn-doped TiO_2_ nanomaterials with different amounts of Sn (1, 2.5, 5, 10, and 15 at%) were prepared by a sol–gel method and characterized by XRD, TG, DTA, EDS, XPS, DRS, SEM, BET, and PL. The photocatalytic activity of the prepared samples was investigated by measuring the degradation of rhodamine B in aqueous solution under UV light. The experimental results indicate that doping with Sn promotes phase transformation from anatase to rutile. The photocatalytic activity of TiO_2_ is influenced by both the heat treatment temperature and the Sn doping concentration. 1% Sn–TiO_2_ exhibits the highest degradation rate at 350 °C and 5% Sn–TiO_2_ exhibits the best photocatalytic activity at 500 °C and 650 °C. The enhancement of the photocatalytic activity can be ascribed to a larger surface area and a better hydration ability, as well as less recombination of the photogenerated pairs.

## Abstract 2

Sn incorporation into TiO_2_ lattices promotes anatase/rutile transformation and Sn–TiO_2_ exhibits better photocatalytic activity at different temperatures.

## Graphical abstract

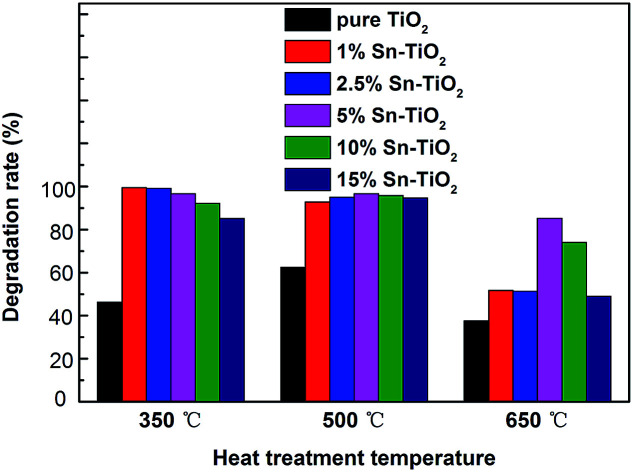

## Introduction

Following industrial development, water pollutants have become a serious environmental problem in the last few decades. In terms of the methods used for waste water treatment, using photocatalysts for decomposing harmful organic pollutants into CO_2_ and H_2_O is one of the most promising strategies. There are several candidate photocatalysts that have been applied to water treatment.^1–4^ Among them, TiO_2_ has been extensively studied owing to its high oxidation, non-toxicity, low cost, and chemical stability.^5–7^ However, pure TiO_2_ is severely restricted in application due to its low solar energy utilization on account of its large bandgap and the fast recombination of photogenerated electrons and holes.^8,9^ Among the several strategies designed to improve the photocatalytic activity of TiO_2_, ion doping is a convenient and effective approach.^10–15^ Among numerous elements, Sn has been reported to be an effective doping resource and is widely used for enhancing the photocatalytic properties of TiO_2_.^16–22^ Sn doping can promote the photocatalytic activity of TiO_2_ due to the fact that Sn^4+^ ions and Ti^4+^ ions have similar radii. Sn^4+^ ions are able to enter into TiO_2_ lattices and create electron traps, resulting in the enhancement of the separation of photogenerated pairs.^23,24^ In addition, the SnO_2_ band is 3.6 eV and its structure is similar to rutile TiO_2_ and, therefore, the recombination of the photogenerated pairs can be inhibited effectively, owing to the combination of TiO_2_ and SnO_2_.^25^

The Sn dopant content plays a key role in the photocatalytic activity of TiO_2_ since there is an optimal concentration. The doping effect cannot be fully exhibited when the doping content is too low. However, Sn ions form recombination centers for the photogenerated pairs when the concentration surpasses the optimal content. Chua *et al.*^26^ prepared Sn-doped TiO_2_ films *via* aerosol assisted chemical vapor deposition and found that the degradation of stearic acid reached the highest value when the Sn/Ti atom ratio was 3.8%. Besides this, heat treatment affects the crystal structure of TiO_2_ significantly and thus annealing temperature is a decisive factor in the photocatalytic performance of TiO_2_. There is usually a transition from an anatase structure to a rutile structure with increasing annealing temperature. The anatase/rutile phase transition temperature is strongly affected by ion doping. The doping of several ions, such as Nd,^27^ La,^28^ Fe,^29^*et al.*, raises the temperature. Meanwhile, doping with Sn reduces the temperature and promotes the transition from anatase to rutile. Alves *et al.*^21^ reported that pure TiO_2_, which demonstrated a single anatase structure, presented excellent photocatalytic activity after annealing at a temperature of 650 °C. At the same temperature, 0.5 wt% Sn-doped TiO_2_, which consisted of 82.4% anatase and 17.6% rutile, showed the highest photocatalytic efficiency.

In view of the above, both the heat treatment temperature and doping concentration have important impacts on the crystal structure and photocatalytic activity of TiO_2_. Therefore, in the present work, pure and Sn-doped TiO_2_ with various amounts of Sn^4+^ ions were synthesized *via* a sol–gel method and the influence of the heat treatment temperature and Sn^4+^ doping concentration on the crystal structure and the photocatalytic activity of TiO_2_ were studied systematically.

## Experimental

### Preparation of pure and Sn-doped TiO_2_ nanomaterials

All the TiO_2_ nanomaterials were prepared *via* a sol–gel method. In a typical preparation of pure TiO_2_, 30 mL tetrabutyl titanate and 60 mL absolute ethanol were added into a beaker. 6 mL deionized water, 15 mL acetic acid, and 45 mL absolute ethanol were added into a pear-shaped funnel. The solution in the pear-shaped funnel was added to the beaker dropwise with continuous stirring. After several hours of aging, the resulting sol formed a gel. The gel was dried at 80 °C for 12 hours and was then annealed for 2 hours at 350 °C, 500 °C, and 650 °C, respectively. Sn-doped TiO_2_ with atom ratios (Sn/Ti) of 1%, 2.5%, 5%, 10%, and 15% was prepared from appropriate amounts of SnCl_4_·5H_2_O being added into a pear-shaped funnel whilst the other experimental conditions were kept equal. For simplicity, *X*% Sn-doped TiO_2_ is labelled as *X*% Sn–TiO_2_ (*X* = 1, 2.5, 5, 10, 15).

### Characterization

X-ray diffraction (XRD) spectra were recorded with a diffractometer (DX-2700, China). Thermogravimetric (TG) and differential thermal analysis (DTA) were performed using a thermal analyzer (STA409PC, Germany). X-ray photoelectron spectra (XPS) were recorded using a spectrometer (XSAM800, Britain) to examine the chemical states. The UV-vis diffused reflectance spectra (DRS) were collected using a spectrophotometer (UV-3600, Japan). The surface morphologies (SEM) and element compositions (EDS) were determined using a field-emission scanning electron microscope (FEI-Inspect F50, USA) equipped with an energy dispersive X-ray spectrometer. Specific surface areas were measured using BET theory on the nitrogen adsorption–desorption data. Photoluminescence (PL) spectra were obtained using a luminescence spectrometer (F-4600, Japan) with a 150 W Xenon lamp as an excitation source.

### Photocatalytic activity experiments

The photocatalytic activity of the prepared TiO_2_ nanomaterials was evaluated from the degradation of rhodamine B (RhB). 300 mL RhB solution (10 mg L^−1^) and 0.3 g TiO_2_ sample were added into a beaker and the suspension was stirred for 30 min in darkness to establish an adsorption–desorption equilibrium between the photocatalysts and RhB molecules. A 250 W high-pressure mercury lamp was employed as a UV light source. The degradation of RhB was monitored by measuring the absorbance of the RhB solution at 553 nm. The degradation rate (*D*) was calculated by the following equation:*D* = (*A*_0_ − *A*_*t*_)/*A*_0_where *A*_0_ and *A*_*t*_ are the initial absorbance and absorbance at time “*t*”, respectively.

## Results and discussion

### XRD analysis

**Fig. 1:**
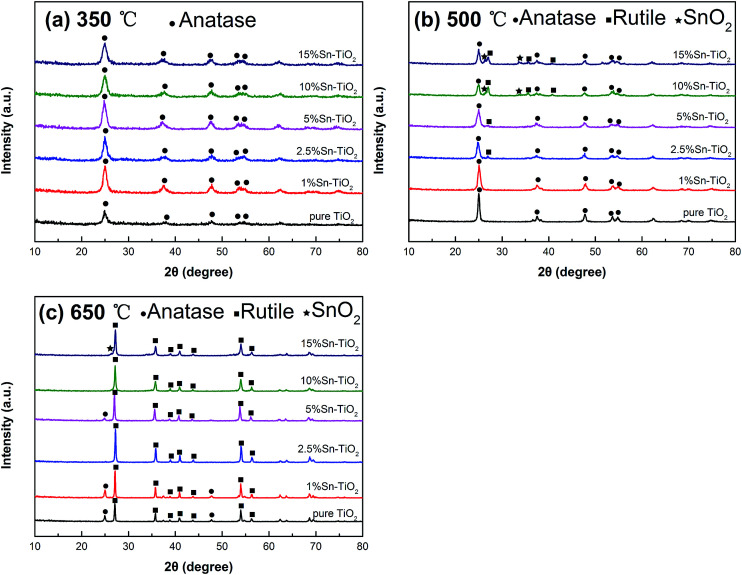
XRD patterns of pure TiO_2_ and Sn–TiO_2_ annealed at 350 °C (a), 500 °C (b), and 650 °C (c).

The XRD patterns of pure TiO_2_ and Sn–TiO_2_ with different concentrations of Sn, annealed at 350 °C, 500 °C, and 650 °C, are shown in Fig. 1. In Fig. 1a, the patterns of all the samples are similar and the peaks can be assigned to the anatase structure. The width of the peaks is relatively wide and the intensity of the peaks is weak, which indicates that both pure TiO_2_ and Sn–TiO_2_ show poor crystallinity at a temperature of 350 °C.

The peak intensity of pure TiO_2_ increases and the peak width narrows as annealing temperature is increased to 500 °C, which suggests that the crystallinity of TiO_2_ increases with increasing temperature. The crystallinity of pure TiO_2_ annealed at 350 °C is determined to be 23.7%, while it is 74.5% for pure TiO_2_ annealed at 500 °C. There is no peak for the rutile structure, which suggests that 500 °C is insufficient for starting the phase transformation from anatase to rutile. 1% Sn–TiO_2_ shows a similar pattern to pure TiO_2_. The rutile peaks appear when the Sn doping concentration reaches 2.5%. The SnO_2_ peaks can be detected in the patterns of 10% Sn–TiO_2_ and 15% Sn–TiO_2_, indicating that SnO_2_ forms in Sn–TiO_2_ when the Sn content is at a sufficiently high level.

It is observed that both anatase and rutile peaks appear in the pattern of pure TiO_2_ at 650 °C, which means that phase transition from anatase to rutile occurs with increasing temperature. The anatase structure peaks become weaker in the Sn–TiO_2_ patterns, and even disappear when the Sn content is 10% and 15%. There is little SnO_2_ forming in the 15% Sn–TiO_2_ sample.

The average crystallite size (*D*) was calculated by the formula *D* = 0.89*λ*/*β* cos *θ* (ref. 30) (where *λ* is the wavelength of Cu Kα, *β* is the full width at half maximum of the XRD peak, and *θ* is the Bragg diffraction angle) and the mass fraction of anatase (*X*_A_) was calculated by the formula *X*_A_ = (1 + 1.26(*I*_R_/*I*_A_))^−1^ (ref. 31) (where *I*_A_ and *I*_R_ are the intensities of the anatase (101) plane and rutile (110) plane) and all the results are shown in Table 1. The crystallite size of pure TiO_2_ increases with increasing temperature. Sn–TiO_2_ shows a smaller crystallite size compared to pure TiO_2_ at the same temperature and the decreasing trend increases with increasing Sn content.

**Table 1:** Crystal structure and crystallite size of all the TiO_2_ nanomaterials

Temperature	Sample	Crystal structure	Crystallite size/nm
350 °C	Pure TiO_2_	Anatase	15.7
	1% Sn–TiO_2_	Anatase	12.0
	2.5% Sn–TiO_2_	Anatase	13.7
	5% Sn–TiO_2_	Anatase	11.3
	10% Sn–TiO_2_	Anatase	10.3
	15% Sn–TiO_2_	Anatase	10.8
500 °C	Pure TiO_2_	Anatase	19.0
	1% Sn–TiO_2_	Anatase	15.0
	2.5% Sn–TiO_2_	Anatase (84.4 wt%)/rutile (15.6 wt%)	14.6/35.6
	5% Sn–TiO_2_	Anatase (89.2 wt%)/rutile (10.8 wt%)	13.5/20.8
	10% Sn–TiO_2_	Anatase (49.7 wt%)/rutile (50.3 wt%)	14.0/15.1
	15% Sn–TiO_2_	Anatase (61.8 wt%)/rutile (38.2 wt%)	17.6/12.1
650 °C	Pure TiO_2_	Anatase (20.2 wt%)/rutile (79.8 wt%)	25.8/36.9
	1% Sn–TiO_2_	Anatase (18.2 wt%)/rutile (81.8 wt%)	27.0/42.3
	2.5% Sn–TiO_2_	Rutile	35.9
	5% Sn–TiO_2_	Anatase (6.8 wt%)/rutile (93.2 wt%)	23.7/34.0
	10% Sn–TiO_2_	Rutile	28.5
	15% Sn–TiO_2_	Rutile	26.5

As shown by the results in Table 1, it is clear that the mass percentage of rutile increases with increasing Sn content, suggesting that the transition from anatase to rutile is promoted and the phase transformation temperature is reduced by Sn doping. There are several explanations for this phenomenon. Tripathi *et al.*^23^ believe that higher surface energy is beneficial for the phase transformation. Anatase of smaller size has a higher surface energy and higher surface area and, therefore, it is easier to start the transformation. This viewpoint is still somewhat disputed. The doping of elements such as La^28^ and Ce^32^ in TiO_2_ always leads to a reduction in crystallite size. However, the phase transformation is inhibited and the transition temperature is evidently enhanced. Ding *et al.*^33^ are convinced that the melting point of M_*X*_O_*Y*_ (M is the doping element) is a key factor in the anatase–rutile phase transformation. The phase transformation will be promoted if the melting point of M_*X*_O_*Y*_ is lower than that of TiO_2_ (1640 °C) and it will be restrained when the melting point of M_*X*_O_*Y*_ is higher than that of TiO_2_. The melting point of SnO_2_ is 1127 °C, which is much lower than TiO_2_, and thus the addition of Sn promotes the phase transformation. Besides this, Kumar *et al.*^34^ and Merhraz *et al.*^35^ hold that the phase structure of SnO_2_ is similar to the rutile structure, which is in favor of the formation of the rutile phase during the phase transition. Consequently, the phase transformation temperature is reduced. In the present study, we believe the relatively low melting point of SnO_2_ and the structural similarity between SnO_2_ and TiO_2_ are propitious to the transition from anatase to rutile. There has been considerable research to demonstrate that TiO_2_ with an anatase structure or anatase/rutile mix structure exhibits excellent photocatalytic activity. From the discussion above, both the heat treatment temperature and Sn doping content affect the phase structure and further affect the photocatalytic activity of TiO_2_. This provides a feasible method to regulate the phase structure and obtain better photocatalytic activity for TiO_2_*via* combining a proper heat treatment temperature and doping amount.

### TG and DTA analyses

**Fig. 2:**
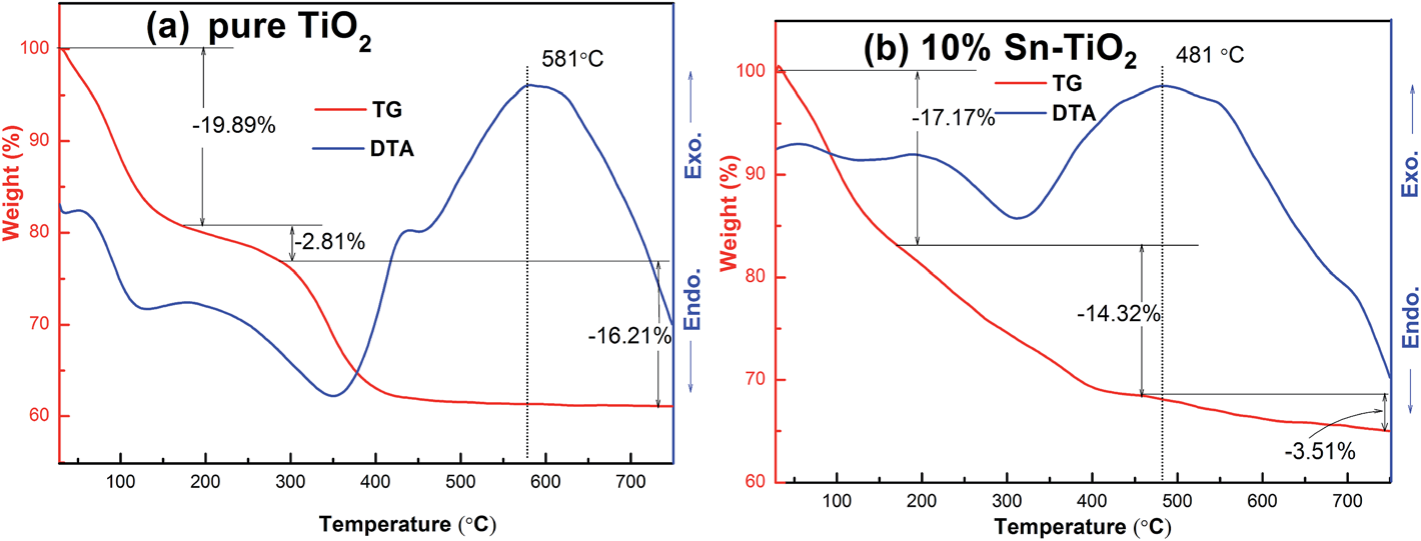
TG and DTA curves for pure TiO_2_ and 10% Sn–TiO_2_.

The thermal behavior and weight loss curves of pure TiO_2_ and 10% Sn–TiO_2_ are shown in Fig. 2. The TG curves of both samples are divided into three steps that are attributed to the evaporation of physically adsorbed water, the combustion of organic compounds, and dehydroxylation, respectively.^20^ However, the total weight loss of 38.91% of 10% Sn–TiO_2_, especially in the second stage with a weight loss of 14.32%, is more than that of the pure TiO_2_ sample, which indicates higher residual organic species losses in 10% Sn–TiO_2_.^23^

As shown by the DTA curves, the exothermic and endothermic peaks of both samples are caused by the decomposition of physically adsorbed water and organic compounds at a temperature below 450 °C. Beyond 581 °C, the exothermic peak in the DTA curve reflects the phase transformation from anatase to rutile TiO_2_. The exothermic peak of pure TiO_2_ at approximately 581 °C can be attributed to both the combustion of organic compounds and the phase transition from anatase to rutile.^7^ Meanwhile, it is noticeable that the exothermic peak of 10% Sn–TiO_2_ is around 481 °C, which is lower than 581 °C, suggesting that the phase transformation temperature of anatase to rutile decreases with Sn doping. It is obvious that the phase transformation from anatase to rutile is promoted by Sn addition, which is in agreement with the XRD analysis in Fig. 1.

### EDS and XPS analyses

**Fig. 3:**
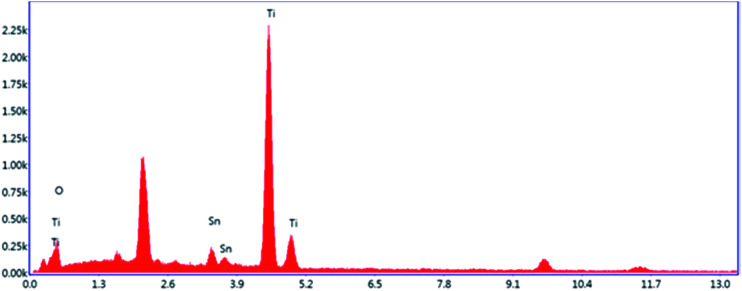
EDS spectrum of 5% Sn–TiO_2_ annealed at 500 °C.

Fig. 3 shows the EDS spectrum of 5% Sn–TiO_2_ annealed at 500 °C. The signals of the elements Ti, O, and Sn appear in the pattern, which confirms that Sn exists in the TiO_2_ sample.

**Fig. 4:**
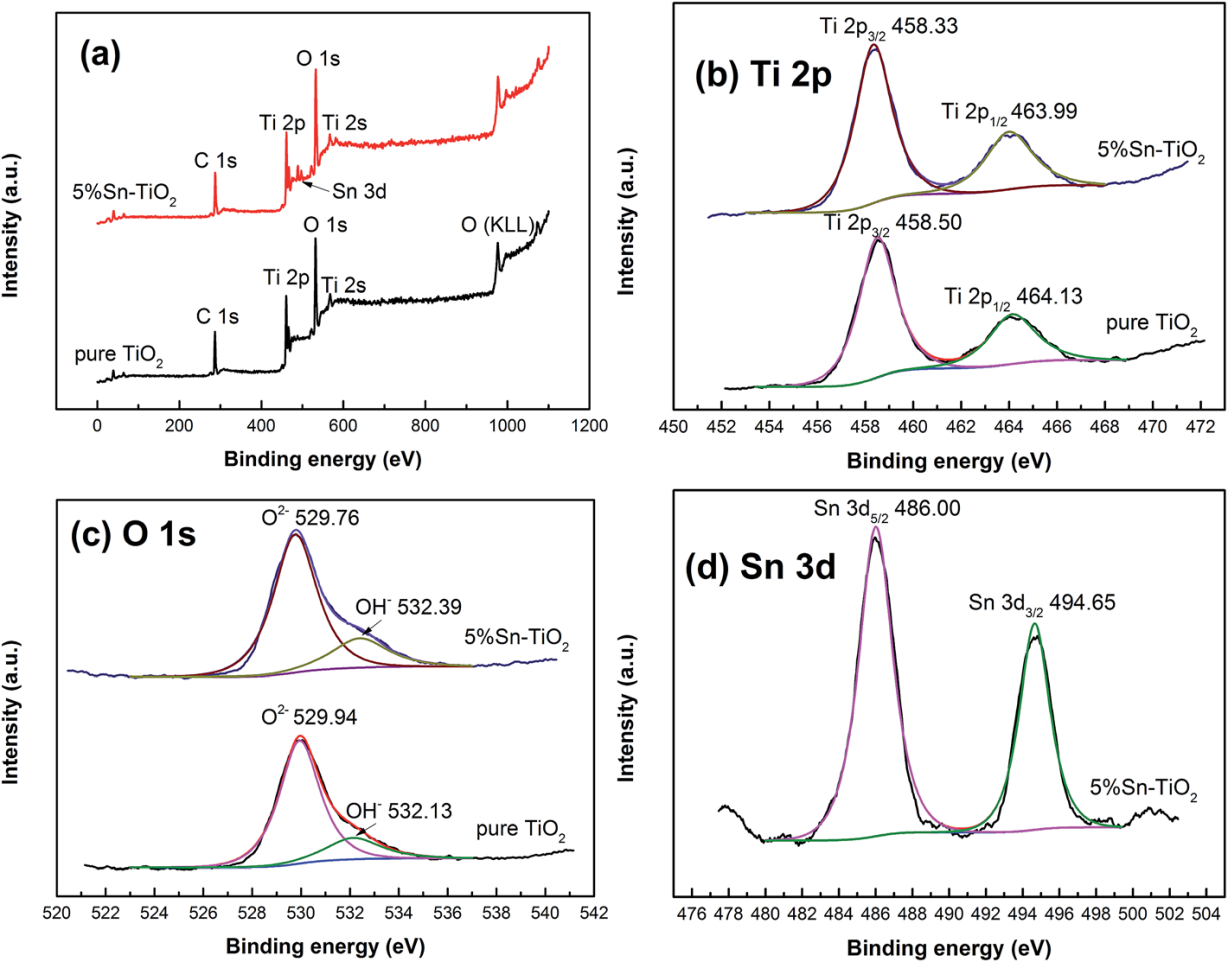
The total spectra of pure TiO_2_ and 5% Sn–TiO_2_ annealed at 500 °C.

To identify the chemical valence states of the existing elements in TiO_2_, XPS measurement was carried out and the results for pure TiO_2_ and 5% Sn–TiO_2_ annealed at 500 °C are shown in Fig. 4. The total spectra of pure TiO_2_ and 5% Sn–TiO_2_ are depicted in Fig. 4a, in which the signals of C, Ti, and O can be detected in pure TiO_2_. Meanwhile, a peak of Sn 3d appears in 5% Sn–TiO_2_, which confirms that Sn exists in TiO_2_*via* doping. The peaks of C 1s can be attributed to oil pollution from the equipment.

The high-resolution spectra of Ti 2p are shown in Fig. 4b. There are two peaks of pure TiO_2_ at 458.33 eV and 463.99 eV, which are assigned to Ti 2p_3/2_ and Ti 2p_1/2_, respectively. The peak position of Ti 2p_3/2_ and Ti 2p_1/2_ suggests that Ti exists as Ti^4+^.^20,36^ Compared to pure TiO_2_, the Ti 2p of 5% Sn–TiO_2_ exhibits a slight positive shift, probably derived from the band bending, which is the consequence of the interaction between Sn, Ti, and O.^37,38^

Fig. 4c shows the high-resolution spectra of the O 1s of pure TiO_2_ and 5% Sn–TiO_2_. The O 1s of pure TiO_2_ consists of two peaks, located at 529.94 eV and 532.13 eV, corresponding to lattice oxygen and surface hydroxyl groups, respectively.^25,37,39^ Similarly, the O 1s of 5% Sn–TiO_2_ can also be divided into two peaks at 529.76 eV and 532.39 eV ascribed to lattice oxygen and surface hydroxyl groups. The ratio of surface hydroxyl groups in pure TiO_2_ is calculated to be 18.9% and it is 21.3% in 5% Sn–TiO_2_. It is obvious that the number of surface hydroxyl groups increases after Sn doping, which is conducive to the photocatalytic process.^25,39^

Fig. 4d depicts the high-resolution spectrum of Sn 3d with two peaks at 486.00 eV and 494.65 eV. These two peaks correspond to Sn 3d_5/2_ and Sn 3d_3/2_, respectively, indicating that the Sn is in the +4 state.^20,25^

### DRS analysis

**Fig. 5:**
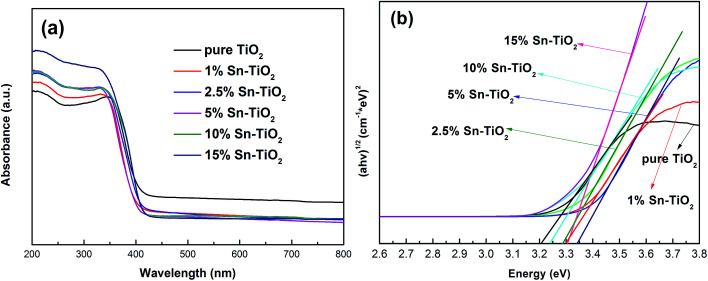
(a) UV-vis diffuse reflectance absorption spectra of pure TiO_2_ and Sn–TiO_2_ annealed at 500 °C. (b) Optical band gap of pure TiO_2_ and Sn–TiO_2_.

The influence of the addition of Sn on the optical properties of TiO_2_ is controversial. Several studies report that a red shift occurs upon Sn doping.^16,17,23,40^ On the contrary, others have concluded that the band gap energy of TiO_2_ increases, thus presenting a blue shift after the addition of Sn.^19,25^Fig. 5 depicts the DRS spectra of pure TiO_2_ and Sn–TiO_2_ annealed at 500 °C. The band gap of TiO_2_ is calculated following the Kubelka–Monk function:^23^(*αhν*)^1/2^ = *A*(*hν* − *E*_g_),where *α* is the absorption coefficient, *hν* is the photon energy, *A* is the proportionality constant, and *E*_g_ is the band gap energy.

The band gap energy of pure TiO_2_ is 3.20 eV, which is in accordance with the theoretical value. The Sn–TiO_2_ samples show a slight blue shift compared to pure TiO_2_. The band gap energy of 1% Sn–TiO_2_, 2.5% Sn–TiO_2_, 5% Sn–TiO_2_, 10% Sn–TiO_2_, and 15% Sn–TiO_2_ are determined to be 3.32 eV, 3.27 eV, 3.31 eV, 3.28 eV, and 3.27 eV, respectively. Obviously, the blue shift in the present work is in accordance with Bhange’s and Zhao’ work.^19,25^ Since size quantization effects come into play when the crystallite size is below 10 nm,^19^ the reason for the blue shift effect on account of Sn doping should not be ascribed to size quantization effects because the crystallite size of Sn-TiO_2_ is from 13.5 to 17.6 nm. The explanation for the blue shift is that the ionic radius of Sn^4+^ is close to that of Ti^4+^, and it is possible for Sn^4+^ ions to substitute Ti^4+^ ions in TiO_2_ lattices, changing the electronic structure of TiO_2_ and forming new energy levels.^19^

### SEM analysis

**Fig. 6:**
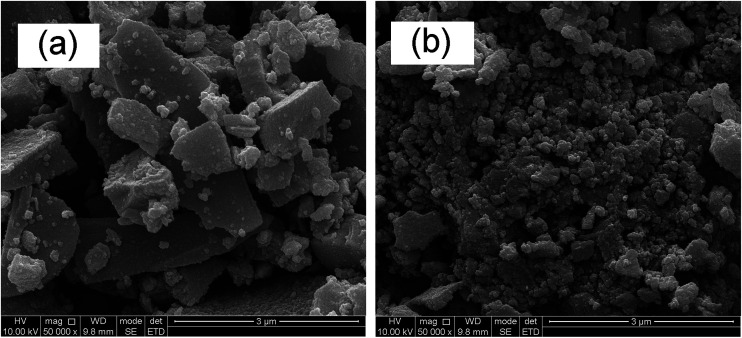
SEM images of pure TiO_2_ (a) and 5% Sn–TiO_2_ (b) annealed at 500 °C.

Fig. 6 depicts the SEM images of pure TiO_2_ (a) and 5% Sn–TiO_2_ (b) annealed at 500 °C. The particles in the pure TiO_2_ sample show an irregular shape and large size distribution. The agglomerated bulks have a diameter in the range of 0.1–3 μm. By comparison, 5% Sn–TiO_2_ consists of smaller particles and exhibits relatively better distribution. The smaller particle size creates more reaction points, which is beneficial for photocatalytic degradation.

### BET analysis

The surface area of the samples was measured using BET theory on the nitrogen adsorption–desorption data and the results are shown in Table 2. The BET surface areas of pure TiO_2_, 1% Sn–TiO_2_, 5% Sn–TiO_2_, and 15% Sn–TiO_2_ annealed at 500 °C are 2.9, 13.8, 54.4, and 61.2 m^2^ g^−1^, respectively. It is obvious that the surface area increases tremendously after adding Sn. A higher Sn dopant level facilitates the enhancement of the surface area. The increased surface area can enhance the absorption of the light source, which contributes to the photocatalytic activity of TiO_2_.^16^

**Table 2:** The BET surface area of the pure TiO_2_ and Sn–TiO_2_ samples annealed at 500 °C

Sample	BET surface area (m^2^ g^−1^)
Pure TiO_2_	2.9
1% Sn–TiO_2_	13.8
5% Sn–TiO_2_	54.4
15% Sn–TiO_2_	61.2

### PL analysis

**Fig. 7:**
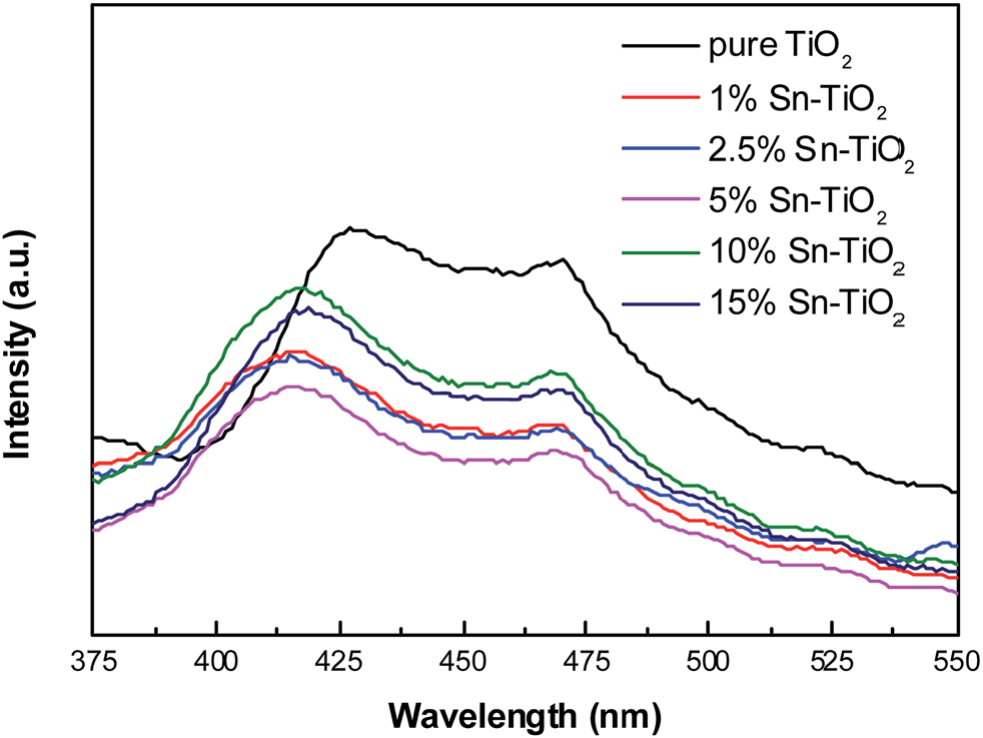
Photoluminescence spectra of pure TiO_2_ and Sn–TiO_2_ annealed at 500 °C.

Since photoluminescence (PL) emission is derived from the recombination of photogenerated electrons and holes, it can therefore provide accurate data of the recombination and separation of photogenerated pairs.^29,41^ The photoluminescence spectra of pure TiO_2_ and Sn–TiO_2_ annealed at 500 °C are shown in Fig. 7. The intensity of the emission spectra is observed to decrease with Sn concentration up to 5%. Sn^4+^ ions act as a trap for photogenerated electrons, thus increasing the separation rate of the photogenerated pairs.^17^ However, the intensity of the PL spectrum increases in the 10% Sn–TiO_2_, which indicates that further Sn doping content is harmful and increases the recombination of the photogenerated pairs. Unexpectedly, the PL spectrum intensity of 15% Sn–TiO_2_ is lower than that of 10% Sn–TiO_2_. Bhange *et al.*^19^ hold that the decrease in PL intensity at high Sn doping levels is derived from the formation of SnO_2_. The photogenerated electrons are able to move to the conduction band of SnO_2_ from the TiO_2_ surface, which improves the separation of the photogenerated pairs.

### Photocatalytic activity

**Fig. 8:**
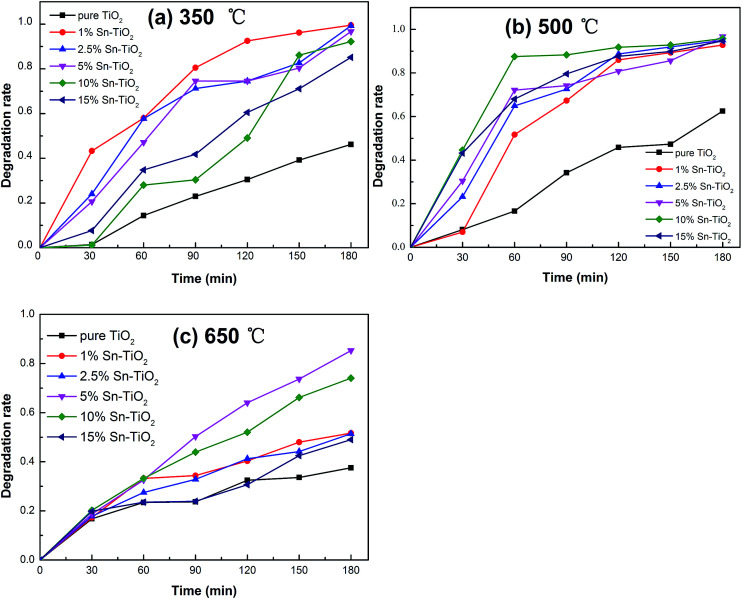
Photocatalytic degradation of RhB for pure TiO_2_ and Sn–TiO_2_ annealed at 350 °C (a), 500 °C (b), and 650 °C (c).

The photocatalytic activity of all the prepared samples was investigated *via* the decomposition of RhB solution. The degradation rates of RhB under UV light over pure TiO_2_ and Sn–TiO_2_ heat treated at 350 °C, 500 °C, and 650 °C are shown in Fig. 8. The prepared TiO_2_ nanomaterials that were heat treated at 350 °C show degradation rates of 46.2%, 99.5%, 99.2%, 96.7%, 92.2%, and 85.1% with an Sn dopant concentration of 0%, 1%, 2.5%, 5%, 10%, and 15% after 180 min. The results reveal that pure TiO_2_ shows relatively low photocatalytic activity at 350 °C. The degradation rate of pure TiO_2_ annealed at 500 °C is 62.5%, which is 1.35 times higher than that of pure TiO_2_ annealed at 350 °C. This enhancement is ascribed to the increase in the degree of crystallinity with rising temperature.^42^

The degradation rates of 1% Sn–TiO_2_, 2.5% Sn–TiO_2_, 5% Sn–TiO_2_, 10% Sn–TiO_2_, and 15% Sn–TiO_2_ annealed at 500 °C are 92.8%, 95.0%, 96.7%, 95.8%, and 94.7%, respectively. All the Sn–TiO_2_ samples show higher degradation rates compared to that of pure TiO_2_, which clearly states that the photocatalytic activity of TiO_2_ improves significantly with Sn doping. From the results of the XRD and BET analyses, it is clear that Sn–TiO_2_ nanomaterials have a smaller crystallite size and larger surface area compared to pure TiO_2_. The increased surface area is in favor of the photocatalytic reaction process, owing to the presence of more reaction spots.^19^ In addition, the lattice expansion caused by the substitution of Ti^4+^ ions with Sn^4+^ ions leads to the formation of more surface defects. The resulting defects capture photogenerated electrons and thus suppress the recombination of the photogenerated pairs effectively,^17^ which is proven by the PL measurement. Besides this, from the XPS results, it is found that more surface hydroxyl groups form on the surface of Sn–TiO_2_, indicating that the hydration ability and the adsorption ability of the RhB molecules are enhanced by Sn doping. Therefore, Sn–TiO_2_ exhibits a higher photocatalytic activity than pure TiO_2_.^25^ Among the samples heat treated at 350 °C, 1% Sn–TiO_2_ shows the best photocatalytic activity. A further increase in the addition of Sn results in a decline in the photocatalytic activity because the excess Sn^4+^ ions act as recombination centers for the photogenerated pairs.^41^ It is worth noting that 5% Sn–TiO_2_ exhibits the best photocatalytic activity, and 10% Sn–TiO_2_ and 15% Sn–TiO_2_ also present high photocatalytic activity. From the XRD results, it is observed that rutile TiO_2_ forms in the 2.5% Sn–TiO_2_, 5% Sn–TiO_2_, 10% Sn–TiO_2_, and 15% Sn–TiO_2_ samples. The mixture of anatase and rutile can promote the transfer of photogenerated electrons, which prolongs the lifetime of the photogenerated pairs and thus improves the photocatalytic activity.^21,24^ SnO_2_ forms in the 10% Sn–TiO_2_ and 15% Sn–TiO_2_ samples and the combination of SnO_2_ with TiO_2_ also has a mixture effect, which is beneficial to the photocatalytic activity.^19,25^

The degradation rates of pure TiO_2_, 1% Sn–TiO_2_, 2.5% Sn–TiO_2_, 5% Sn–TiO_2_, 10% Sn–TiO_2_, and 15% Sn–TiO_2_ heat treated at 650 °C are 37.5%, 51.7%, 51.4%, 85.2%, 74.0%, and 49.0%, respectively. All the samples show lower photocatalytic activity compared to those treated at 500 °C, owing to the high rutile content (Fig. 1c). It is well known that rutile TiO_2_ exhibits lower photocatalytic activity because of its poor hydroxylation and oxygen absorption. As a result, excess rutile leads to lower photocatalytic activity.^24,43^

**Fig. 9:**
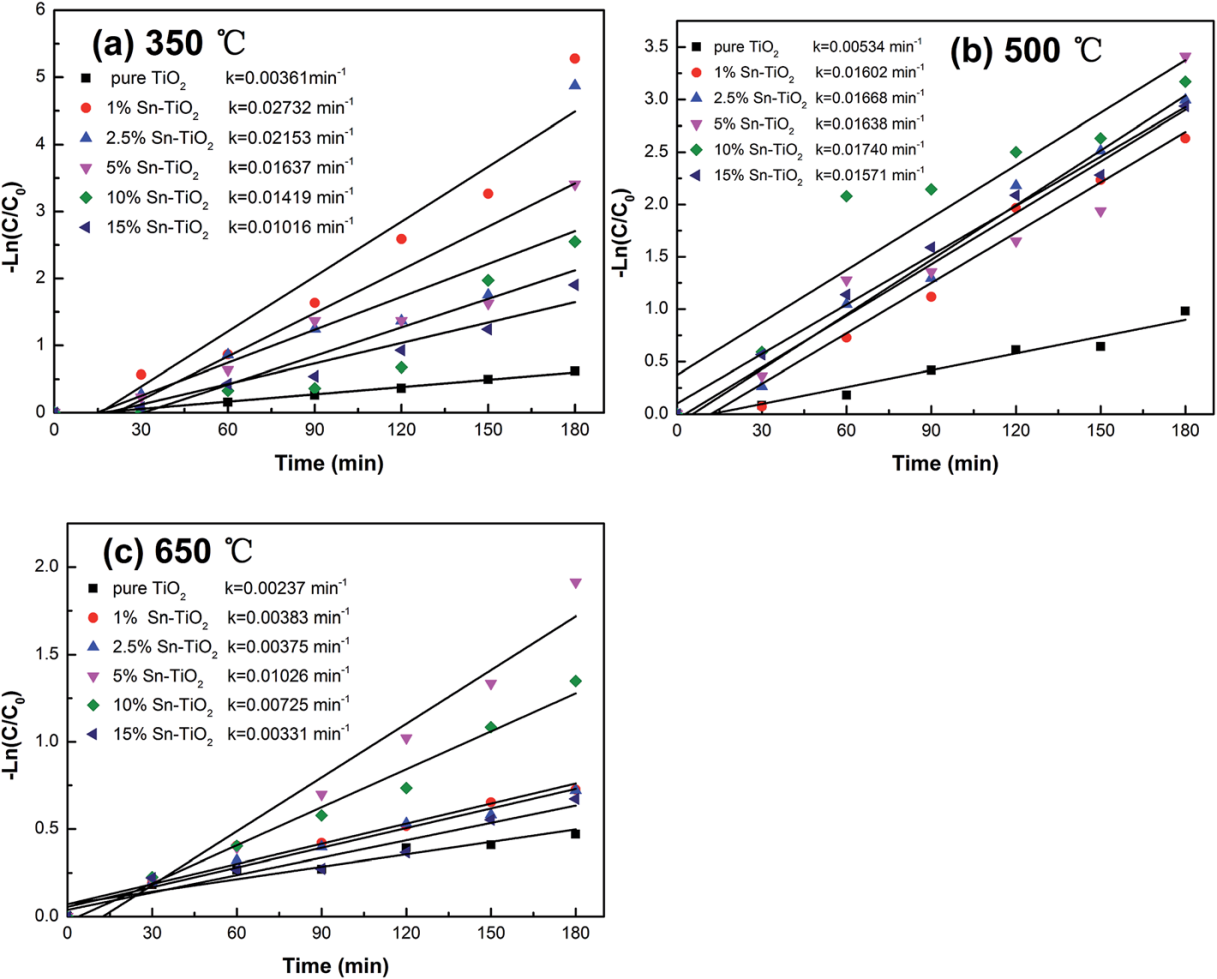
First-order reaction rate constant *k* against reaction time for different photocatalysts annealed at 350 °C (a), 500 °C (b), and 650 °C (c).

The kinetics of the photocatalytic degradation of RhB can be described by a first order kinetics model and the reaction rate constant *k* can be calculated from the following equation:^44^*kt* = −ln(*C*_*t*_/*C*_0_)where *t* is the reaction time, *C*_*t*_ is the concentration of RhB at *t* time, and *C*_0_ is the initial concentration. The graphs of ln(*C*_*t*_/*C*_0_) *versus* reaction time (*t*) for all the samples are shown in Fig. 9, which demonstrate that both the heat treat temperature and Sn content affect the photocatalytic activity of TiO_2_ significantly. The reaction constant of 1% Sn–TiO_2_ annealed at 350 °C is determined to be 0.027 min^−1^, which is 7.5 times higher than that of pure TiO_2_ at 350 °C. The reaction constant of 10% Sn–TiO_2_ annealed at 500 °C (0.017 min^−1^) is 3.4 times higher than that of pure TiO_2_ (0.005 min^−1^) and the reaction constant of 5% Sn–TiO_2_ annealed at 650 °C (0.010 min^−1^) is 5 times higher than that of pure TiO_2_ (0.002 min^−1^) at the same temperature. The results indicate that the addition of Sn evidently improves the photocatalytic activity of TiO_2_, especially when the heat treatment temperatures are 350 °C and 650 °C.

## Conclusion

In summary, we reported a simple sol–gel method for the synthesis of pure TiO_2_ and Sn–TiO_2_. The prepared TiO_2_ nanomaterials were characterized by XRD, TG, DTA, EDS, XPS, DRS, SEM, BET, and PL. The results reveal that all the samples form anatase after calcining at 350 °C, while rutile appears in Sn–TiO_2_ with high dopant levels at temperatures up to 500 °C and rutile is dominant at 650 °C. The addition of Sn promotes the phase transformation from anatase to rutile. The photocatalytic test results show that both the heat treatment temperature and Sn dopant concentration affect the photocatalytic activity of TiO_2_. 1% Sn–TiO_2_ shows the highest degradation rate at 350 °C and 5% Sn–TiO_2_ exhibits the highest degradation rates at 500 °C and 650 °C.

## Conflicts of interest

There are no conflicts to declare.

## Supplementary Material
